# Evaluating the efficacy of intraoperative NIRS cutoff values in detecting spinal cord ischemia during surgery

**DOI:** 10.1007/s10877-025-01331-w

**Published:** 2025-07-22

**Authors:** Sebastian Zinn, Nia Joseph, Travis Stanley CreveCoeur, Roman M. Sniecinski, Paul S. García

**Affiliations:** 1 Department of Anesthesiology, Columbia University Medical Center, New York, USA; 2 Department of Anesthesiology, Emory University, Atlanta, USA; 3 Department of Neurosurgery, Columbia University Medical Center, New York, USA; 4 Department of Anesthesiology, Intensive Care Medicine and Pain Therapy, Goethe University Frankfurt, University Hospital, Frankfurt am Main, Germany

**Keywords:** Thoracoabdominal aortic aneurysm, Near-infrared spectroscopy, Spinal cord ischemia, Neurological outcomes, Tissue oxygenation monitoring

## Abstract

Purpose: Paralysis is a serious complication of surgeries that interferes with the blood supply of the anterior spinal cord, with rates of spinal cord injury (SCI) from approximately 1% in general spine surgeries to 4–40% following thoracoabdominal aortic aneurysm (TAAA) repair. Near-infrared spectroscopy (NIRS) provides a non-invasive, real-time method for monitoring tissue oxygenation, largely unaffected by anesthetics. Given the heightened risk of neurologic injury during TAAA repair, this procedure is used to evaluate the effectiveness of standard regional spinal oxygen saturation (rSpO₂) cutoff values in predicting neurological outcomes. Methods: This retrospective study analyzed 25 patients undergoing open TAAA repair. NIRS data were recorded at the ischemic site and a reference location throughout surgery. Neurological outcomes were assessed postoperatively based on paralysis, hemiparesis, or extremity weakness. After excluding eight patients due to poor signal quality, 17 patients were included. NIRS values at six key time points were compared between outcome groups. Bayesian statistics assessed the relationship between significant NIRS “drops” (< 80% of baseline) and neurological outcomes. Results: Seven patients exhibited new neurological deficits (4 temporary). No credible association was found between intraoperative NIRS drops and postoperative neurological outcomes at any analyzed time point. A moderate effect was observed at the end of surgery (Hedges’ g = − 1.21), suggesting a potential difference between groups, although the Bayesian credible interval included zero (posterior mean = − 0.82, 94% HDI [− 1.8, 0.18]). Conclusions: In this limited cohort, intraoperative NIRS cutoff values did not significantly correlate with postoperative neurological deficits following TAAA repair. Postoperative NIRS monitoring may be more informative for detecting spinal cord ischemia and preventing paralysis.

**Supplementary Information:**

The online version contains supplementary material available at 10.1007/s10877-025-01331-w.

## Introduction

Paralysis is a severe and feared complication associated with surgeries involving the vessels supplying the anterior spinal cord. While the incidence of spinal cord injury (SCI) after spine surgery is rare (approximately 1%), it increases to between 4% and 40% following thoracoabdominal aortic aneurysm (TAAA) repair. Due to the higher risk of postoperative neurological injury during TAAA repair, it serves as an appropriate model for evaluating monitoring technologies designed to alert physicians to potential malperfusion of the spinal cord. The incidence of SCI after TAAA repair is influenced by perfusion techniques and type of aneurysm, with the greatest risk occurring in Crawford types I and II [1, 2]. Given the high risk of SCI during TAAA repair, various perfusion strategies have been developed to prevent spinal cord injury - like left heart bypass (LHB), which can reduce the risk of paralysis by more than 50% [3, 4]. However, even with LHB, decreases in distal tissue oxygenation do still occur. This underscores the need for effective intraoperative monitoring of spinal cord perfusion. Near-infrared spectroscopy (NIRS) is a non-invasive method for monitoring tissue oxygenation in real-time using transcutaneous probes. NIRS provides continuous perfusion data, making it a potentially valuable tool for detecting malperfusion during surgery [5, 6]. In addition to being non-invasive, NIRS is mostly unaffected by anesthetics, suggesting a potential benefit over traditional intraoperative neuromonitoring techniques (e.g., SSEPs and MEPs) [7, 8]. 

In this small preliminary study, we explore the potential for intraoperative monitoring of regional tissue oxygenation with NIRS technology to predict adverse neurologic outcomes. Previously, these types of monitors have been studied for predicting adverse cognitive outcomes after cardiac or aortic surgery [9, 10] but evidence is lacking for clear “cut-off” values that can be used to determine clinically significant intraoperative changes in regional spinal oxygen saturation (rSpO₂). Regarding NIRS monitoring of cerebral perfusion, tissue oxygen saturation values below 60% rSpO₂ or relative decreases of more than 20% from baseline are often considered critical [11–13]. However, it is unclear whether these thresholds are applicable to distal tissue measurements in the context of aortic surgery or spinal ischemia. Furthermore, the relevance of the timing of perfusion changes during surgery in predicting postoperative neurological complications remains uncertain. Therefore, we hypothesized that intraoperative NIRS monitoring of regional oxygen saturation during thoracoabdominal aortic (TAAA) repair, using a 20% drop from baseline as a cutoff, correlates with postoperative neurological deficits, providing a potential marker for spinal cord malperfusion.

## Methods

The study was approved by the Institutional Review Board (IRB) of Emory University (Approval Number: IRB00060168) and was conducted in accordance with the ethical standards of the Declaration of Helsinki. Between 2012 and 2014, a total of 25 patients over the age of 18 undergoing open surgical repair of thoracoabdominal aortic aneurysm (TAAA) with enhanced perfusion technique (deep hypothermic circulatory arrest, DHCA; cardiopulmonary bypass, CPB; left heart bypass, LHB) were enrolled in this study. Exclusion criteria included emergency surgeries and prior spinal cord injuries. NIRS data were collected using the Somanetics INVOS 5100 C (Coviden, Mansfield MA, USA).

After anesthesia induction, NIRS pads were placed near the 10th thoracic vertebra, as the area at risk for ischemia. The other electrodes were placed in the upper area between the scapulae, close to the vertebrae– in an area not at risk for ischemia. The pads were placed on either the tips of the spinous processes or the opposing lamina. NIRS data were continuously collected at 30-second intervals throughout the operation. The choice of enhanced perfusion technique was based on surgeon preference.

Signal pre-processing was performed using MATLAB 2023b (Mathworks). A sliding median filter with a 4-minute window was applied to smooth out transient fluctuations and reduce noise in the NIRS signals. Patients with incomplete or poor-quality NIRS data were excluded from the final analysis. The mean value of 10 flanking values was taken for each time point of interest.

### Statistics

A 2 × 2 contingency table was constructed to compare the presence of significant NIRS drops (≥ 20% from baseline) with neurological outcomes. Neurological outcome was determined by postoperative clinical examination during the hospital stay and classified as true if permanent or transient neurological symptoms of paralysis, hemiparesis, or weakness of the extremities were noted by the clinical team. There was no clinical follow up on long-term neurological persistent weaknesses or fragility.

For binary outcomes, we used a Bayesian logistic regression model (logit link) to estimate the probability of postoperative paralysis as a function of a >20% NIRS drop, between-signal disagreement, and their interaction with perfusion type. All regression coefficients were assigned weakly informative priors: Normal(0, 2). Posterior distributions provide probabilistic estimates of the associations, including interaction effects, at key surgical time points.

To compare relative changes in physiological signals between patients with and without postoperative paralysis, we used a Bayesian linear regression model. The standardized outcome variable was modeled as a function of group membership, with priors specified as: mu0 ~ Normal(0, 2), delta ~ Normal(0, 1), and sigma ~ HalfNormal(1). In this model, delta represents the standardized group difference. Posterior distributions were estimated using the No-U-Turn Sampler (2,000 draws, 1,000 tuning steps, target_accept = 0.95). The posterior mean of delta reflects the estimated effect size, integrating prior beliefs with observed data. A 94% highest density interval (HDI) that excludes zero indicates strong evidence for a group difference, while an HDI overlapping zero suggests no conclusive effect.

For the logistic model, posterior estimates of the regression coefficients represent changes in the log-odds of paralysis; positive values indicate increased risk. All Bayesian models were implemented in Python using PyMC (version 5.23.0) [14]. To quantify effect size between groups, Hedge’s g was calculated as a frequentist reference using Python (Version 3.8, scipy.stats).

## Results

From the 25 patients initially enrolled, 8 were excluded due to poor signal quality, missing data, and/or withdrawal of IC, resulting in a final analysis of 17 patients (10 females; mean age 57.4 ± 11.9 years). The patients either received deep hypothermic circulatory arrest (DHCA) (*n* = 10) or cardiopulmonary bypass (CPB)(*n* = 7) to enhance perfusion. Seven patients demonstrated a new postoperative neurologic deficit (4 temporary)(Supplementary Fig. 3). Patients with paralysis had a median duration of perfusion technique of 165.5 [range: 56.5-223.5] min, while patients without paralysis had a median of 182.0 [53.0-224-5] min. We calculated a possible mild effect with a Hedges’ g and a posterior mean of 1.8 [HDI − 0.066 − 3.7] (Supplement Fig. 1). No clear difference in paralysis risk between DHCA and CPB technique was observed (Supplementary Fig. 2).

**Fig. 1:**
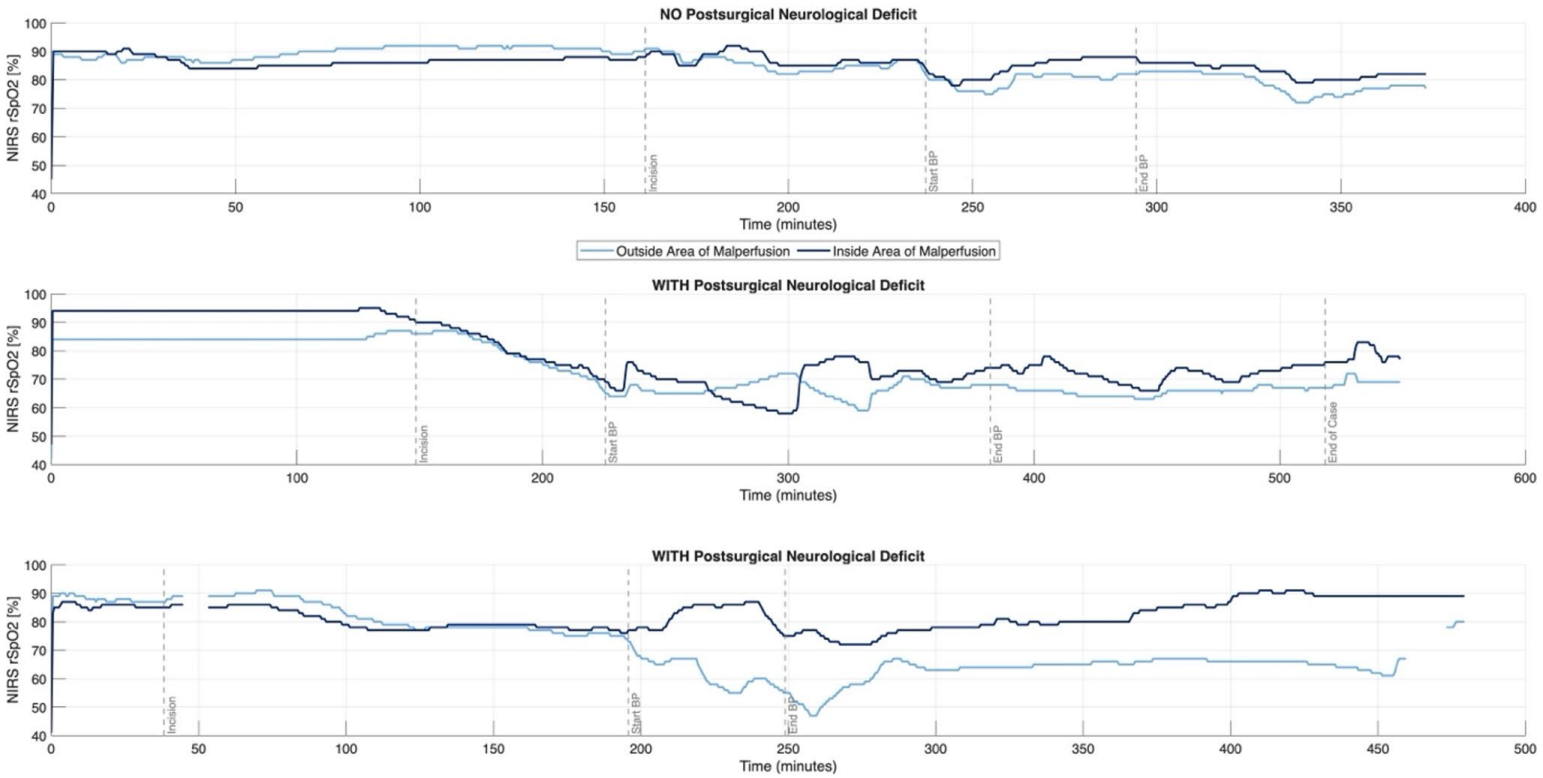
Representative plots of the NIRS time series are shown for a patient without postoperative neurologic deficits (**A**) and for two separate patients with postoperative neurologic deficits (1**B**&**C**). Vertical lines indicate the events of interest

### Comparison of signal data

**Fig. 2:**
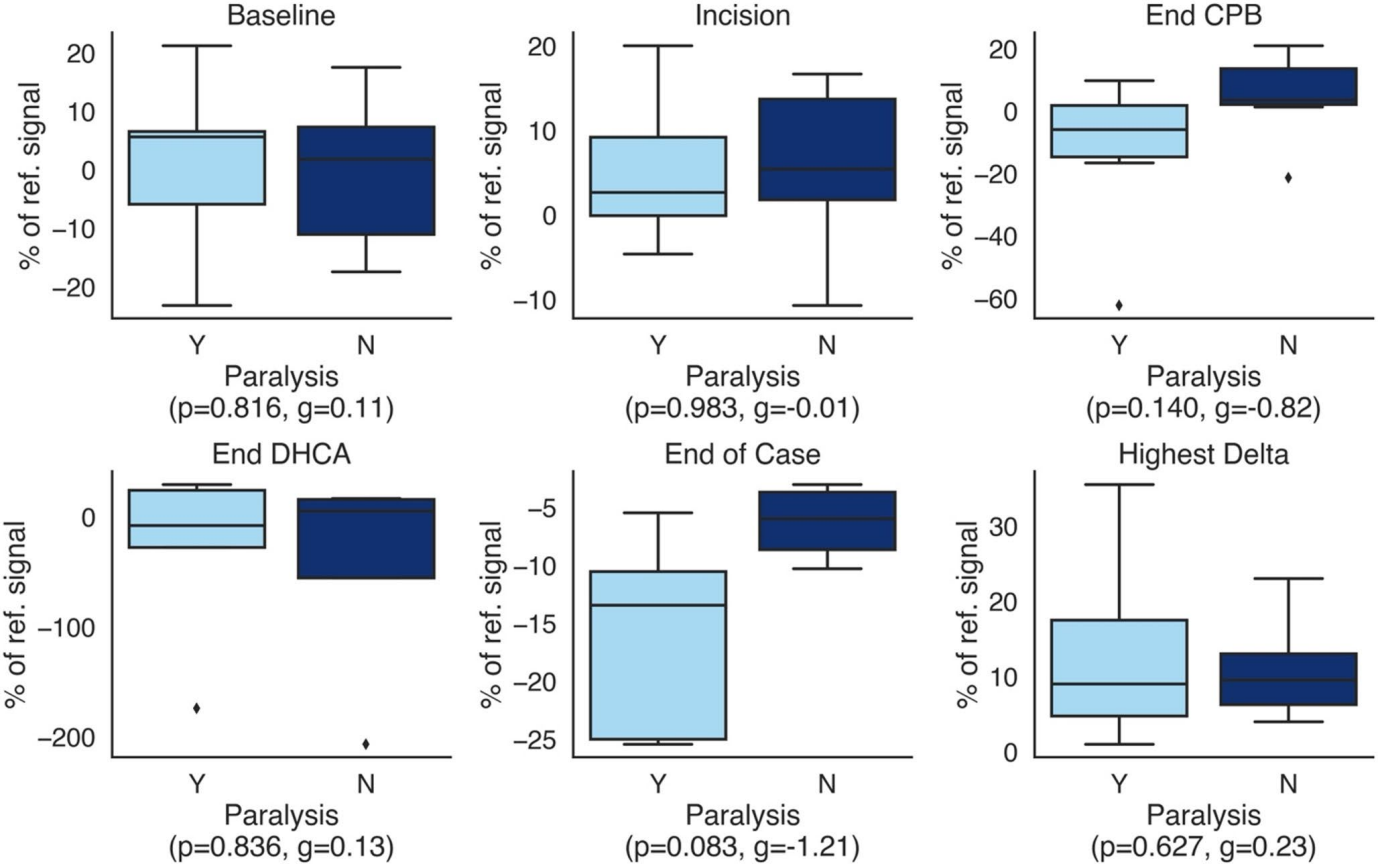
Comparison of signal data at key time points during surgery Box plots showing the distribution of relative differences between NIRS signals at key time points for patients with and without postoperative neurological deficits. The central line represents the median value; the box spans the interquartile range (25th to 75th percentile), and the whiskers extend to 1.5 times the IQR. Outliers are depicted as individual data points. There was no significant difference between the two groups for any time point in t-testing. For the last time point during surgery, “End of Case” a Hedges’ g indicates an effect with g = -1,2 Statistics: t-test and effect size calculation with Hedges’ g. Small (d > 0.2), medium (d > 0.5), and large (d > 0.8)

The relative change between the two NIRS signals are compared. Representative plots of NIRS time-series are shown in Fig. 1 for a patient without postoperative neurologic deficits (1 A) and for two separate patients with postoperative neurologic deficits (1B&C). We selected key time points for analysis: baseline (obtained after induction), incision, end of perfusion technique, end of case (either skin closure or the end of NIRS measurement). We also examined the highest relative difference between the two NIRS channels signals independent of time (Fig. 2) and during the time of perfusion technique used (Supplement Fig. 1). There was no significant difference between those with versus those without neurologic injury for any key time point. Also, the drop during perfusion technique did not result in a relevant effect (g = 0.23; posterior mean 0.95 [HDI − 0.78 − 2.7] Also, patients with neurologic injury did not on average demonstrate an overall larger maximum difference between the two NIRS signals during surgery. The two groups are best separated at the “End of Case” time point although the Hedges’ g indicated an effect size (g = 1,21) between the groups, that was not statistically significant (Fig. 2) and the HDI included zero for all time points (Supplementary Fig. 4).

### Group comparison related to cut-off values

We also examined whether a “cut-off” threshold coincided with the occurrence of postoperative neurologic dysfunction. For each key time point, a positive threshold crossing was scored if the rSPO2 signal fell below 80% of reference. We not only used the NIRS signal at baseline as the reference but also the other NIRS signal (Table 1). For each combination of time-point and references, patients were grouped based on whether they fell below this threshold or not. In our Bayesian logistic regression model was no association of positive threshold crossing with neurologic injury for any comparison.

**Table 1:** Group comparison related to cut-off values with a bayesian logistic regression model. The analysis examined whether falling below a threshold of 80% compared to the reference signal coincided with post-surgical neurologic deficits. there was no conclusive effect

TimePoint	Parameter	Posterior Mean	HDI Lower (3%)	HDI Upper (97%)
Incision	NIRS below base line (NIRS bbl)	0.000	-3.759	3.663
Incision	NIRS bbl x DHCA Interaction	− 0.007	-3.752	3.735
Incision	Signal Disagreement	− 0.099	-2.927	2.516
End of Perfusion Technique	NIRS below base line (NIRS bbl)	− 0.623	-3.660	2.280
End of Perfusion Technique	NIRS bbl x DHCA Interaction	− 0.651	-3.569	2.344
End of Perfusion Technique	Signal Disagreement	0.079	-2.522	2.823
Skin Closure	NIRS below base line (NIRS bbl)	− 0.005	-3.863	3.671
Skin Closure	NIRS bbl x DHCA Interaction	0.0350	-3.777	3.750
Skin Closure	Signal Disagreement	0.0830	-2.716	2.861

## Discussion

Our preliminary examination of intraoperative NIRS monitoring of regional oxygen saturation during thoracoabdominal aortic aneurysm (TAAA) repair does not appear to reliably predict post-operative neurological deficits when comparing NIRS signals at key intraoperative time-points or when using a thresholding method. Because of the variability in signal fidelity and the small size of our study, we remain optimistic that refinement of these techniques could aid in clinical decision-making for surgeries that involve potential mal-perfusion of the anterior spinal cord. This metered optimism comes from observing a modest trend in our limited data set, focused on the differences between NIRS signals at the end of the case.

### Comparison with previous studies

Hypoperfusion of the spinal cord and resultant hypoxia are known risk factors for paralysis after TAAA surgery. Specifically, ischemia of the artery of Adamkiewicz is often discussed. Etz et al., in their study of thoracoabdominal aortic aneurysms in pig models, discovered that within days of blood flow compromise, spinal cord preservation was achieved through a collateral network of blood vessels [15]. Therefore, perhaps regional tissue ischemia does not consistently lead to permanent paralysis and that collateral blood supply during recovery is an important factor. In another study by Etz et al., they conducted a clinical series to measure lumbar and thoracic regional oxygenation during TAAA in humans. Interestingly, they found no significant difference in thoracic collateral network oxygenation between patients who did and did not experience paraplegia. Still, they found some predictive value in patients that had perfusion discrepancies *before* distal perfusion initiation [16]. Although Etz et al. did not suggest that preoperative perfusion issues contribute to spinal cord injury. This pattern raises the possibility that early intraoperative perfusion deficits, before full bypass or distal perfusion is established, could be clinically significant. Of note, that study involved open, endovascular, and hybrid procedures, whereas our study only involved open repair [16]. 

The most sensitive method to immediately identify and address spinal cord dysfunction involves utilizing somatosensory evoked potentials (SSEPs) and motor evoked potentials (MEPs) and these techniques have been shown to correlate with NIRS data. Currently, studies directly correlating evoked potentials with NIRS parameters on their influence of neurological outcomes are lacking [8, 17]. Neurophysiologic activity can be reduced during administration of inhaled anesthetics and are only measured intraoperatively. NIRS has potential benefits over MEPs and SSEPs in that it is immune to pharmacologic influence and can be utilized during the postoperative period. Our data trends towards distinguishing between neurologic injury by NIRS measurements at the “end of case” time point. Thus suggesting a possible role for NIRS measurements in the early post-operative period to detect hypoperfusion due to either (a) failure in fidelity of collateral blood supply, (b) post-surgical edema, or (c) both.

### Limitations

One limitation of this study is that the dataset is over ten years old. The device used for data acquisition remains commercially available and is widely used in current clinical practice. Although the underlying sensor technology has not fundamentally changed, the retrospective nature and age of the dataset may need to be considered with respect to present-day clinical workflows, surgical techniques, and patient populations. Neurological outcomes were assessed during the hospital stay through daily clinical examinations by the clinical care team; however, no standardized long-term neurological follow-up was available after discharge. Intraoperative neuromonitoring (e.g., EMG) and objective postoperative testing were not performed. Therefore, delayed or subtle impairments may have been missed, and some deficits classified as persistent may have improved over time. To address this uncertainty, both transient and persistent motor deficits were grouped as clinically meaningful acute postoperative neurological complications.

Prior studies on brain tissue oxygenation have found NIRS data can correlate with postoperative complications [9, 18]. These studies focused more broadly on defects in brain perfusion like hemorrhage or ischemia. For example, in carotid endarterectomy, NIRS has been found to be a valuable monitoring tool, with a 12.3% decrease in regional oxygen saturation identified as a reliable threshold for intraoperative cerebral hypoperfusion [19]. NIRS measurement of spine surrounding tissue instead is also only a surrogate for spinal cord perfusion itself. This must be considered when compared to cerebral NIRS, where brain tissue is measured more directly. Even though the physiological consideration that reduced tissue oxygenation in the spinal cord increases the probability of a neurological disorder is evident, valid thresholds, defined by tissue type and neurological outcome, for perioperative use are still lacking [13]. Another limitation of this study is that not all intraoperative hypoperfusion events always lead to ischemia or some kind of reperfusion injury. Heterogeneity is often seen in other cerebrovascular surgeries, such as carotid endarterectomy [20], where the usual autoregulation is impaired resulting in neural injury.

Future studies should examine the association of neurologic injury with the specific types and locations of dysregulation of the neurovascular unit (e.g., thoracic cord, lumbar cord, cerebral cortex). Another potential limiting aspect of this study is comparing the turbulence of flow and possible dissection within the aneurysms themselves, which may contribute to spinal cord hypoperfusion at baseline [21, 22]. The effects of fluid dynamics in TAAA repair would limit the extrapolation of these results to spinal cord perfusion.

## Conclusion

In conclusion, while our study did not find statistically significant correlations between NIRS-measured regional tissue perfusion and postoperative neurological outcomes, the observed trends highlight the need for further research to establish reliable thresholds and improve the use of NIRS monitoring in surgeries and post-operative care that impact spinal cord perfusion. Studies on spinal hypoperfusion should document not only sufficient group sizes but also other correlates of spinal cord injury in detail. We therefore recommend detailed hemodynamic measurements, standardized baseline measurements and sensor locations, intraoperative EMG measurements, postoperative continuation of NIRS measurements for 24 h, and a sufficiently long clinical follow-up for future studies. Due to the effect sizes presented here, which are based on a limited sample, we suggest a follow-up study and invite interested readers and investigators to collaborate on this.

## Electronic supplementary material

Below is the link to the electronic supplementary material.

Supplementary Material 1

## Data Availability

The model data that support the findings of this study are not openly available due to reasons of sensitivity and are available from the corresponding author upon reasonable request..
