# Supplementary material for: Evaluating the efficacy of intraoperative NIRS cutoff values in detecting spinal cord ischemia during surgery
Source: J Clin Monit Comput. 2025 Jul 22;40(2):309–15. doi: 10.1007/s10877-025-01331-w (PMC13053335; doi:10.1007/s10877-025-01331-w)
Supplement: Supplementary file 1 — Supplementary Material 1 [file 10877_2025_1331_MOESM1_ESM.docx]

Supplementary material

**
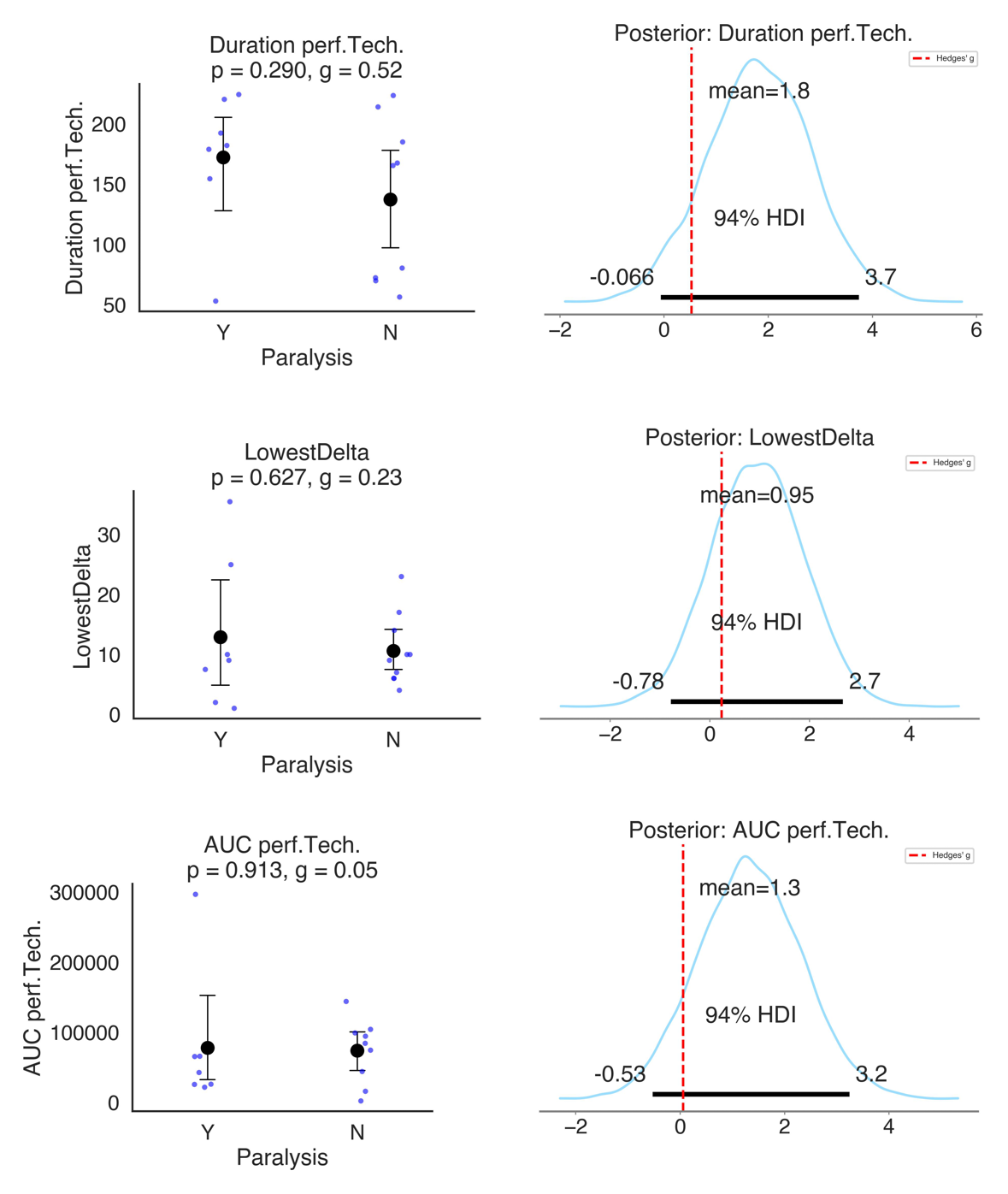
**

Supplement Figure 1: Comparison of the duration of perfusion, the lowest delta during the perfusion and the Area Under the Curve between the two signal channels. Left panels show classical group comparisons using dot plots with group means and 95% confidence intervals. P-values (Welch’s t-test) and Hedges’ g are reported as effect size estimates. Right panels show the Bayesian posterior distributions for the group difference (delta) with posterior mean (solid line) and 94% highest density interval (HDI). A dashed red line indicates Hedges’ g for visual comparison.


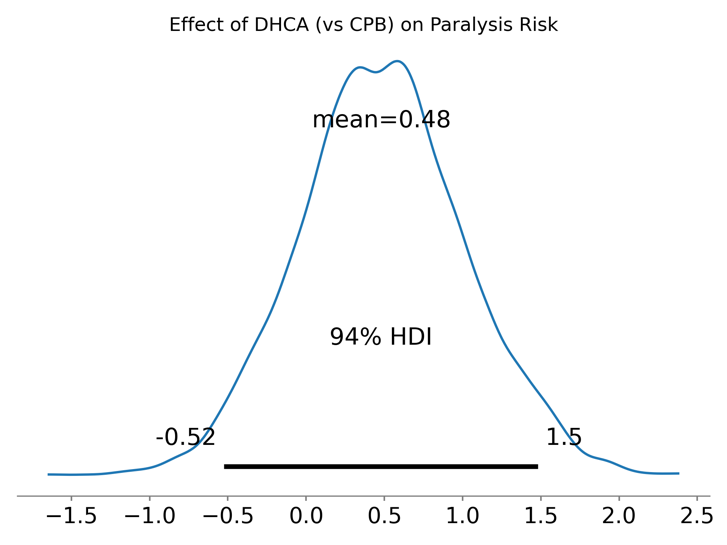


Supplement Figure 2: Posterior distribution of the estimated effect of perfusion strategy (DHCA vs. CPB) on the probability of postoperative paralysis. The curve represents the posterior distribution of the regression coefficient from a Bayesian logistic regression model. The posterior mean is 0.48, suggesting a possible increased risk of paralysis associated with DHCA. The horizontal bar indicates the 94% highest density interval (HDI), which ranges from −0.52 to 1.5. As the HDI includes zero, the estimate does not provide strong credibility for a difference in risk between the two perfusion strategies.

**
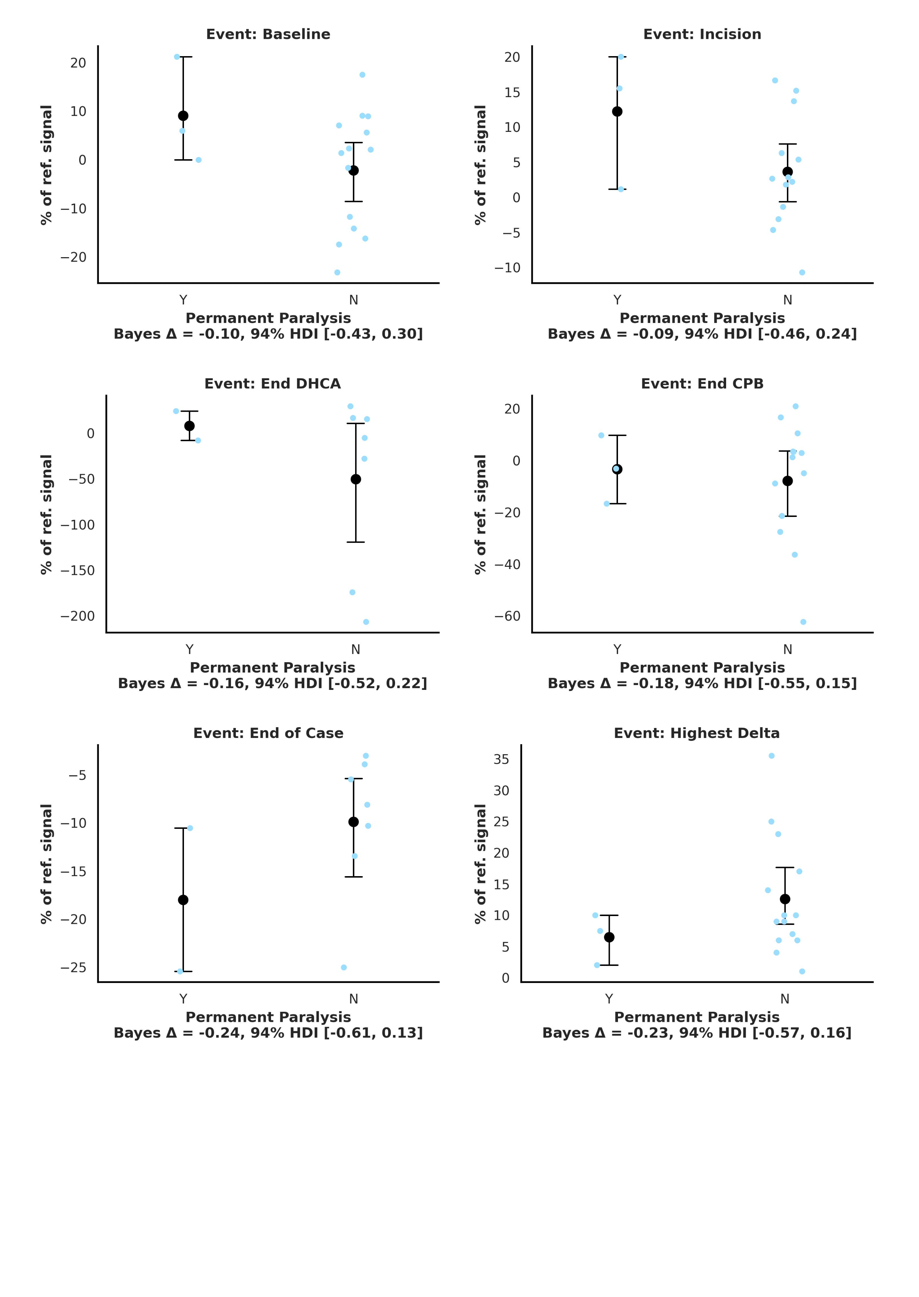
**

Supplement Figure 3: Comparison of signal data at key time points during surgery for patients with permanent paralysis at discharge.

Point plots display individual data points. The error bars indicate the 95% CI. Bayesian estimate of the group difference (Δ) with its 94% highest density interval (HDI). There was no credible difference between the two groups at any time point as the HD crosses zero at all time points.

**
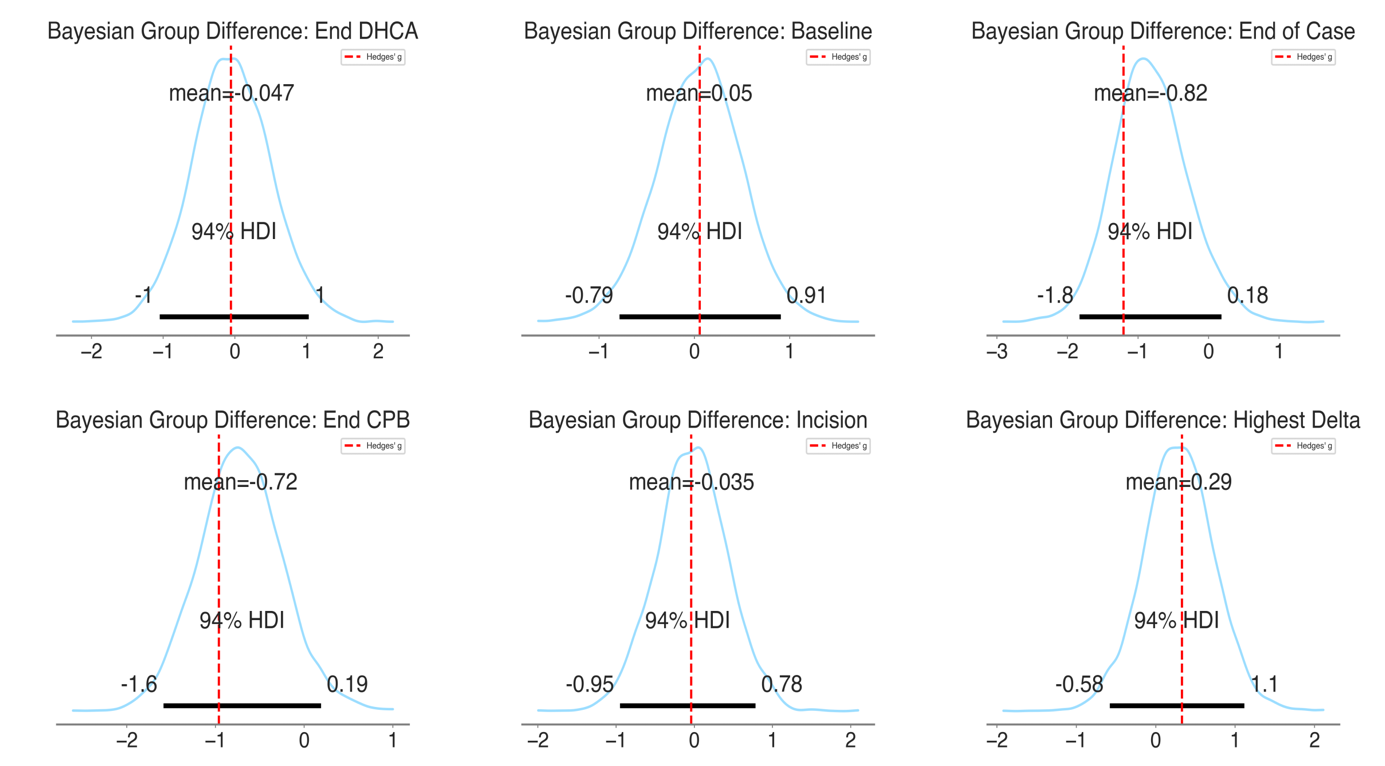
**

Supplement Figure 4: Posterior distributions of group differences in interhemispheric signal asymmetry between patients with and without permanent paralysis at six key intraoperative time points. Each panel displays the posterior distribution of the group difference (delta), with the posterior mean and 94% highest density interval (HDI) indicated. Vertical dashed lines mark Hedges’ g as a frequentist effect size for comparison. In this analysis, the use of weakly informative priors (Normal(0, 1)) and standardized data results in posterior means that closely approximate the corresponding Hedges’ g, reflecting strong agreement between the Bayesian and frequentist estimates when data are moderately informative.
